# Correlation between low handgrip strength and metabolic syndrome in older adults: a systematic review

**DOI:** 10.20945/2359-4292-2023-0026

**Published:** 2024-05-07

**Authors:** Joana da Costa d'Avila, Talel Georges Moreira El Nabbout, Hayfa Georges Moreira El Nabbout, Aline dos Santos Silva, Antonio Carlos Barbosa Ramos, Eliana Rosa da Fonseca, Aluana Santana Carlos, Rodrigo de Azeredo Siqueira

**Affiliations:** 1 Universidade Iguaçu Laboratório de Pesquisa Pré-clínica Grupo de Pesquisa em Biologia Experimental e Humana Nova Iguaçu RJ Brasil Grupo de Pesquisa em Biologia Experimental e Humana, Laboratório de Pesquisa Pré-clínica, Universidade Iguaçu (UNIG), Nova Iguaçu, RJ, Brasil; 2 Universidade Federal do Rio de Janeiro Rio de Janeiro RJ Brasil Sistema de Bibliotecas e Informação da Universidade Federal do Rio de Janeiro (UFRJ), Rio de Janeiro, RJ, Brasil

**Keywords:** Metabolic syndrome, central obesity, insulin resistance, aging, handgrip strength

## Abstract

Muscle weakness has been associated to insulin resistance and metabolic syndrome in the general population. However, it is still unclear whether this association is maintained in older adults. This study investigated correlations between low handgrip strength (HGS) and metabolic syndrome, or some of its components, in older adults through a systematic review of the literature. Searches were conducted in the Virtual Health Library Regional Portal, Scopus, Cochrane, Embase, MEDLINE/PubMed, SciELO, and Web of Science databases for relevant studies investigating muscle weakness (measured by hand dynamometer) and metabolic syndrome or its components in older adult populations, published up to September 2023. From the 2050 references initially identified, 20 studies, comprising a total of 31,264 older adults of both genders, completely met the inclusion/exclusion criteria. Eighteen studies showed that lower HGS was associated with metabolic syndrome or some of its risk factors, such as abdominal obesity, hyperglycemia, insulin resistance, dyslipidemia, or high blood pressure. Two studies found that older men with high blood pressure had increased HGS. Most studies included in this systematic review revealed a significant correlation between reduced HGS and metabolic syndrome or some of its components, especially abdominal obesity and insulin resistance. We conclude that below-average HGS can be associated with metabolic syndrome in older adults.

## INTRODUCTION

Metabolic syndrome is a combination of three or more risk factors including abdominal obesity, high blood pressure, hyperglycemia, and dyslipidemia, that increases the risk of cardiovascular disease, stroke, and diabetes, and contributes to a decline in longevity (1-3). The prevalence of metabolic syndrome in Brazil is high compared to that of other countries (4,5), and evidence indicates that incidence increases with age (5-7). Abnormalities, such as central obesity and hyperglycemia, are predictive of mobility limitations (6), and have been associated with low muscle strength and worse physical performance in older adults (8,9).

Handgrip strength (HGS), measured with a handheld dynamometer, is a simple, non-invasive, and convenient tool to assess upper extremity isometric strength (10). HGS has been shown to predict mortality in various populations of different ages (11), and cross-sectional and longitudinal relationships with cardiovascular risk, functional impairment, and multimorbidity have been described (12-16). Recent studies have proposed normalizing HGS by body weight or body mass index (BMI) to better define subgroup-specific handgrip weakness, referred to as relative HGS as opposed to absolute HGS. Low absolute and relative HGS consistently correlate with metabolic syndrome in the general population (17); however, there is no consensus about this correlation in the older adult population specifically.

HGS has been proposed as a biomarker of frailty and a powerful predictor of future morbidity and mortality (18). HGS naturally decreases with age and is usually lower in women; however, the relationship between chronological age and HGS is nonlinear and can vary between populations (18,19). Therefore, the present study aimed to systematically review the literature on the correlations between low muscular strength measured by a handheld dynamometer and metabolic syndrome, or some of its components, specifically in the older adult population.

## METHODS

### Protocol

Before performing the review, a protocol was created by R.A.S. and J.C.D. to define the objective, search strategy, criteria for inclusion and exclusion of studies, data to be extracted, and quality assessment. The hypothesis investigated was whether muscle weakness, as measured with a handheld dynamometer, is associated with metabolic syndrome or any of its components in older adults. This systematic review was conducted and reported according to the Preferred Reporting Items for Systematic Reviews and Meta-Analysis (PRISMA) statement (20).

### Eligibility criteria

We included original quantitative studies that examined associations between HGS and metabolic syndrome or its risk factors. We used the following *a priori* criteria in the synthesis: (i) complete original articles that reported (ii) observational studies including cross-sectional, longitudinal, case-control, cohort, or prospective studies; (iii) with analysis of prevalence, correlations, and associations that allowed simultaneous control by covariables or based on regression models, with descriptive, odds ratio, and relative risk data; (iv) that reported HGS (kg) measured with a handheld dynamometer, whether on the dominant hand or both hands, expressed alone (HGS) or combined with body weight or BMI (relative HGS); (v) with metabolic syndrome or its components as outcomes (high blood pressure, elevated waist circumference, high triglycerides, low HDL cholesterol levels, high fasting blood glucose, and/or insulin resistance); which included (vi) community-dwelling older adults or clinical patients (above 60 years old) from any ethnic group.

We excluded studies without a clear definition of metabolic syndrome components or metabolic syndrome diagnostic criteria.

### Search strategy

The search was conducted in the Virtual Health Library Regional Portal, Scopus, Cochrane, Embase, MEDLINE/PubMed, SciELO, and Web of Science databases for articles published in Spanish, English, or Portuguese, until 12 March 2023. Afterward, we updated the search to include articles published until September 2023. The descriptors and related terms were searched by sectors: population (Sarcopenia, Muscle Strength, Handgrip Strength, Aged, Elderly, Senescence, Aging); interventions (Muscle Strength, Hand Dynamometer); and Outcomes (Metabolic Syndrome, Insulin Resistance, Cardiovascular Syndrome, Sarcopenia, Muscle Strength Dynamometer). The complete search strategy is fully described in the Supplementary material.

### Study selection and data extraction

First, duplicate articles were discarded. Two independent authors (H.G.M.N. and A.C.B.R.J.) selected the titles and abstracts based on the eligibility criteria. Two other authors (T.G.M.N. and A.S.S.) reviewed the full texts. Interrater agreement was 87.5% (Cohen's k = 0.484). Discrepancies in the full-text screening results were discussed with a third author (J.C.D.). Disagreement was resolved by consensus meetings among all authors. We used Rayyan, a free web application that primarily supports systematic review and meta-analysis research (21).

The following information was extracted: the first author's surname, publication year, country of study, study design, sample size, proportion of males and females, average age, HGS method, dynamometer model, metabolic syndrome outcome, and major findings associating HGS to metabolic syndrome or its components. Correlation coefficients, odds ratios, mean differences, P values, and linear regression statistics were extracted from the full-text studies for inclusion in the synthesis. All information was recorded on a standardized data collection form and checked by all authors.

### Quality assessment

The methodological quality of the included studies was assessed by two authors (A.S.C. and J.C.D.) using the Appraisal tool for Cross-Sectional Studies (AXIS) (22). AXIS comprises 20 questions evaluating study design, methods, quality, and risk of bias in cross-sectional studies. Studies were scored according to multiple criteria with a final score ranging between 0 and 20. The score was converted into a percentage for comparability. AXIS ratings ≥75%, 50%-74%, and <50% were considered good, moderate, and poor quality, respectively (see supplementary material for scoring template and criteria).

## RESULTS

### Literature searches

**Figure 1:**
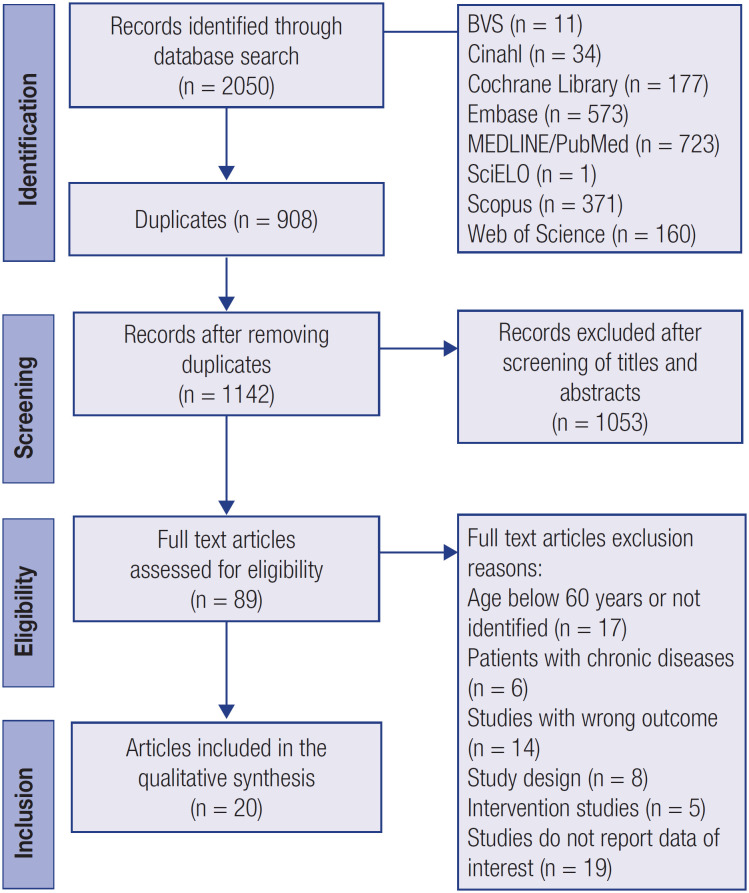
Flow diagram of data search and selection.

We initially identified 2,050 potential articles. After removing duplicates and reviewing the titles and abstracts, 1,961 articles were excluded. Then, 89 articles underwent a full-text review according to the eligibility criteria. Full-text screening led to the exclusion of 69 articles. In total, 20 studies, comprising a total of 31,264 older adults of both genders, completely met the inclusion criteria and were selected for the qualitative synthesis of this systematic review. Figure 1 shows the selection process scheme.

### Study characteristics

All the studies included older adults with mean age above 60 years. Two studies included very old participants (above 85 years) in separate groups (23,24). Most studies included older adults of both genders in fairly similar proportions, except two (25,26). Most studies included non-institutionalized community-dwelling older adults, except two that recruited nursing home elder (27) and hospitalized individuals (28). Two studies included only type 2 diabetic patients (29,30) and one included female patients who visited a healthcare center for preventive purposes, without severe disease (25).

Of the 20 studies included, 17 were cross-sectional and 3 were longitudinal. Publications dates ranged from 2007 to 2023, and articles came from 11 different countries. Twelve studies were considered good and two were considered moderate by the AXIS rating. The sample size was above 2,000 in 7 studies, between 1,000 and 2,000 in 2 studies, between 500 and 1,000 in 4 studies, and below 500 in 7 studies. Characteristics of the included studies are summarized in Table 1.

**Table 1:** Study characteristics

First author, publication year	Country of study	Study design	Sample size (female %)	Average age (years)	Hgs technique (dynamometer model)	Mets outcome	Major finding associating hgs with mets
Biblioni, 2018 [31]	Spain	Cross-sectional	380 (54)	70	HGS (digital, TKK 5401)	WC	Lower HGS in men with abdominal obesity (36.9 kg) compared to non obese (43.3 kg, p=0.017)
Chen, 2021 [32]	Singapore	Cross-sectional	292 (57.9)	74	HGS (digital, A5401)	Modified ATP III for Asians	Higher prevalence of low HGS in MetS frail (76.2%) compared to robust (43.8%, p=0.02)
Chun, 2019 [38]	Korea	Cross-sectional	2709 (53)	68	Relative HGS (digital, Takei, TKK 5401)	Modified ATP III for Asians	Low relative HGS associated to MetS [OR 4.194 (2.985–5.892)]
Escribà-Salvans, 2022 [27]	Spain	Cross-sectional	104 (84.6)	86	HGS (Jamar Digital)	obesity	Obesity was associated with low muscle strength [OR = 0.14 (0.03 ‐ 0.60), p = 0.008]
Gong, 2022 [28]	China	Cross-sectional	84 (51.2)	75	HGS (digital, CAMRY EH101)	Lipoprotein subfractions	HGS was negatively correlated with TG (r = -0.256, p = 0.019)
Joo, 2022 [26]	Korea	Cross-sectional	206 (0)	65-80	Relative HGS (digital, Takei TKK 5401)	HOMA-IR	The prevalence of IR decreased in higher relative HGS. Increase in OR of having IR with lower rHGS [2.82 (1.10–7.21)]
Kawamoto, 2016 [37]	Japan	Cross-sectional	1679 (55.8)	70	Relative HGS (not informed)	Modified ATP III for Asians	Low relative HGS associated with MetS components [WC r=-0.5 (p<0.001); HDL r=0.2 (p<0.001)]
Kim, 2020 [39]	Korea	Cross-sectional	2451 (54.5)	63	Relative HGS (digital, Takei, TKK 5401)	cardiometabolic markers	Low relative HGS associated with MetS components [HOMA-IR r=-0.17, WC -0.4 and high HDL r=0.14, (p<0.001)]
Kimura, 2021 [29]	Japan	Cross-sectional	157 (40.1)	70	HGS (Smedley)	BP in T2DM patients	SBP and PP negatively associated with HGS in women [β = −0.14 (−0.21, −0.08)] DBP positively associated with HGD in men [β = 0.20 (0.03, 0.37)]
Leite, 2023 [30]	Brazil	Cross-sectional	138 (66.7)	68	HGS (hydraulic, Jamar)	central obesity, DM	Lower HGS in participants with central obesity [waist to hip ratio decreased HGS by 41.1% (β= -0.19; t = −3.70; p < 0.001)]
Lin, 2021 [33]	Taiwan	Longitudinal	3739 (57.2)	76	HGS (digital, Takei, TKK5101)	WC	Weaker HGS in old men with higher WC [β = −0.108 (p< 0.001)]
Merchant, 2020 [40]	Singapore	Cross-sectional	722 (55)	71	Relative HGS (digital, A5401)	Modified ATP III for Asians	Higher relative HGS significantly associated with lower odds of having MetS [OR 0.51 (0.43–0.61) and 0.13 (0.07–0.24), p<0.01]
Pérez-Sousa, 2020 [41]	Colombia	Cross-sectional	1571 (60)	70	Relative HGS (digital, Takei)	WC, fasting glucose	Low relative HGS associated with WC [β = −0.069 (−0.082, −0.057), p < 0.001], indirect effect mediated by hyperglycemia [β = −0.002 (−0.004 to −0.001), p < 0.001]
Sayer, 2007 [42]	United Kingdom	Cross-sectional	2677 (52)	66	HGS (Jamar)	ATP III and IDF	Low HGS associated with increased odds of having MetS [OR 1.18 (1.07, 1.30) p<0.001]
Souza Moreira, 2022 [36]	Brazil	Longitudinal	7905 (60)	63	HGS (hydraulic, SAEHAN)	obesity, WC	Overweight was inversely association with muscle weakness [OR 0.66 (0.52–0.83 in men)], obesity was inversely associated with muscle weakness [OR 0.49 (0.31–0.78) in men; OR 0.69 (0.52–0.92 in women)]
Stessman, 2017 [23]	Israel	Longitudinal	2304 (nd)	70-90	HGS (5001 Grip-A)	DM	Prevalence of DM higher in low HGS aged (27%, p=0.004). Survival rates significantly lower in participants with low HGS (p<0.0001)
Taekema, 2011 [24]	Netherlands	Cross-sectional	550 (66)	85	HGS (digital, Jamar)	MAP, SBP, DBP	Higher SBP associated with higher HGS in the oldest [Adjusted β = 0.05, p<0.01]
Tong, 2022 [34]	China	Cross-sectional	251 (60.2)	≥60	HGS (hydraulic, Jamar 5030J1)	IDF	Lower HGS in men with MetS (37.8±7.1Kg) compared to without MetS (42.5±7.5 Kg, p=0.004)
Waqas, 2022 [35]	Netherlands	Cross-sectional	2744 (66)	74	HGS (hydraulic, Fabrication Enterprises)	hyperglycemia	Hyperglycemia biomarker (advanced glycation end-products) was inversely associated with HGS [β −0.051 (95% CI −0.075, −0.026)]
Zhang, 2021 [19]	China	Cross-sectional	601 (100)	67	Relative HGS (digital, Takei TKK5401)	Modified ATP III for Asians	Increased prevalence of MetS in the lowest relative HGS quartile [OR 1.239 (1.052–2.124), p<0.001]

Abbreviations: ATP III-National Cholesterol Education Program-Adult Treatment Panel III; BP-blood pressure; DM-diabetes mellitus; DPB-diastolic blood pressure; HDL-high density lipoprotein cholesterol; HGS-handgrip strength; IDF-Internation Diabetes Federation; MAP-mean arterial pressure; MetS-metabolic syndrome; nd-not determined; PP-pulse pressure; Q1-lower quartil; SBP-systolic blood pressure; SD-standard deviation; T2D-type 2 diabetes; TG-triglycerides; WC-waist circunference. OR (95%CI)-Odds ratio (95% confidence interval). r-correlation coefficient.

### Handgrip strength measures

Twelve studies used digital hand dynamometers, four used hydraulic dynamometers, and four did not state the dynamometer type. Regarding the HGS evaluation, twelve studies used absolute HGS, seven used relative HGS (normalized to body weight), and one used both. Four studies informed the cutoff values for low HGS: <30 kg for men and <20 kg for women in Bibiloni and cols. (31); <28 kg for men and <18 kg for women in Chen and cols. (32); <30 kg for males and <20 kg for females in Lin and cols. (33); and <28 kg for males and <18 kg for females in Tong and cols. (34). Two studies used the EWGSOP2 (European Working Group on Sarcopenia in Older People) criteria of weak muscle strength of HGS as <27 kg for men and <16 kg for women (27,35). The Brazilian Longitudinal Study of Aging (ELSI-Brazil), a nationally representative sample of older Brazilian adults, considered muscle weakness as HGS values lower than the 20th percentile by age group and sex (36). Leite and cols. considered absolute HGS as the sum of the maximum readings from both hands (30).

The remaining articles studied associations between HGS and some metabolic syndrome parameters or stratified HGS values in groups: Kawamoto and cols. correlated HGS values with metabolic syndrome parameters (37). Some studies divided the participants into quintile groups according to grip strength, grip strength/body weight, and grip strength/BMI for each sex (38), into quartile groups according to grip strength for sex (23,25), or into tertiles of relative HGS (T1, ≤1.387 kg/BMI; T2, 1.388-1.613 kg/BMI; and T3, ≥1.614 kg/BMI) (26). Kim and cols. used age-adjusted partial correlation coefficients to estimate the relationship between clinical parameters and absolute or relative HGS (39). Kimura and cols. performed unadjusted and adjusted regression analyses for HGS and blood pressure (29). Merchant and cols. calculated the odds ratio and beta-coefficients of individual HGS indices on risk of metabolic syndrome (40). Pérez-Souza and cols. conducted a mediation analysis to determine the indirect effect of fasting glucose levels on the relationship between abdominal obesity and relative HGS (41).

Sayer and cols. analyzed relationships between grip strength and components of the metabolic syndrome using partial correlation coefficients and analysis of variance. Using multiple linear regression, the authors calculated sex-specific standard deviation scores for HGS and metabolic syndrome parameters and investigated the relationships between a standard deviation decrease in grip strength and each standard deviation score. Logistic regression models yielded odds ratios (and 95% confidence intervals) for each definition of the metabolic syndrome per standard deviation decrease in HGS (42). Stessman and cols. categorized HGS according to sex-specific quartiles within each age group (70, 78, 85, and 90 years) to compare old and very old populations, with low grip strength defined as the lowest quartile (0%-25%) (23). Taekema and cols. analyzed the association between blood pressure and HGS by linear regression models adjusted for gender, height, and weight (24).

### Metabolic syndrome outcomes

From the 20 studies included, six had metabolic syndrome as the primary outcome, diagnosed with criteria established by the National Cholesterol Education Program – Third Adult Treatment Panel (ATP III) (43). The ATP III definition of metabolic syndrome requires three or more of the following five disorders: elevated waist circumference (>102 cm in men and >88 cm in women), hypertriglyceridemia (>150 mg/dL or reported use of triglyceride-lowering drugs), low HDL cholesterol level (<40 mg/dL in men and <50 mg/dL in women or reported use of drugs that increase HDL concentrations), high blood pressure (systolic blood pressure, SBP >130 mmHg and/or diastolic blood pressure >85 mmHg and/or pharmacological treatment), and elevated fasting glucose (>100 mg/dL and/or pharmacological treatment). Of these studies, five used ATP III adjusted for the Asian population, which considers lower cutoff points of waist circumference (>90 cm in men and >80 cm in women).

The other studies used one or more components of metabolic syndrome as outcomes: three studies evaluated blood pressure (24,29,42); eight studies evaluated abdominal obesity by waist circumference (27,30,31,33,36,39,41,42); four studies evaluated insulin resistance (23,26,39,41); and three studies evaluated diabetic patients (23,29,30). One study used skin autofluorescence as the primary outcome, a biomarker of advanced glycated end-products (AGE) resulting from chronic hyperglycemia, and found it inversely associated with HGS. In addition, the highest quartile of AGE biomarker comprised a significantly higher number of individuals with diabetes and weak HGS (35). Two studies included patients with prevalent chronic diseases, such as type 2 diabetes, hypertension, hyperlipidemia, myocardial infarction/angina, cerebrovascular accident, osteoarthritis, or osteoporosis (29,30).

### Associations of low HGS with metabolic syndrome or its components

Most studies included in this systematic review found an inverse correlation between HGS and metabolic syndrome or its components. Older adult patients with insulin resistance consistently presented low HGS in different studies (23,26,35,36). Sayer and cols. found that decreased HGS was associated with higher blood pressure in a population-based study of older men and women (42). On the other hand, Taekema and cols. found that higher blood pressure was associated with higher HGS only in the oldest subjects (>85 years), while in middle-aged adults, blood pressure and HGS were not significantly associated (24). Tong and cols. found an association between high HGS and high SBP in older men (34), and Kimura and cols. found that diabetic male patients with high blood pressure had higher HGS (29).

Six studies showed that reduced HGS was associated with metabolic syndrome (25,32,37,38,40,42). Chen and cols. included only participants with metabolic syndrome. The authors found a higher prevalence of reduced HGS in the metabolic syndrome frail, who were also older, with an increased prevalence of diabetes and low HDL compared to that of metabolic syndrome robust participants (32). Zhang and cols. found a higher prevalence of metabolic syndrome in women with low relative HGS (25). Chun and Kawamoto found inverse correlations between relative HGS and metabolic syndrome (37,38). Merchant and cols. and Sayer and cols. found that relative HGS was associated with decreased odds of having metabolic syndrome (40,42). However, Tong and cols. found a positive correlation between HGS and metabolic syndrome in older men (34).

Chun and cols. analyzed the relationship between HGS and relative HGS (grip strength/body weight or grip strength/BMI) and metabolic syndrome using the data of 1,273 men and 1,436 women aged 60-80 years. Absolute HGS was not associated with metabolic syndrome, whereas relative HGS measures were inversely associated with metabolic syndrome in both sexes. Between relative HGS measures tested, HGS divided by body weight was better associated with metabolic syndrome (38).

Eight studies found consistent associations between central obesity and muscle weakness measured by HGS (27,29,30,33,35,36,39,41). Kim and cols. showed that relative HGS was inversely correlated with waist circumference and insulin resistance and the authors reported a direct correlation between HGS and HDL cholesterol levels (39). High odds ratio for various chronic diseases in the lowest relative HGS tertile were observed for both sexes, while high odds for hyperlipidemia was observed only in women (39). Pérez-Sousa and cols. showed that waist circumference and higher levels of fasting glucose were inversely correlated with relative HGS (41).

Sayer and cols. found that decreased HGS was associated with increased waist circumference and high triglyceride levels, high blood pressure, hyperglycemia, and insulin resistance (42). Joo and cols. also reported an increased prevalence of insulin resistance among the lower HGS subjects (26). Gong and cols. used lipoproteins as primary outcomes and found an inverse correlation between HGS and triglycerides and other lipoprotein subfractions (28).

One study used skin autofluorescence as the primary outcome, a biomarker of advanced glycated end-products (AGE) resulting from chronic hyperglycemia, and found it inversely associated with HGS. Also, the highest quartile of AGE biomarker contained a significantly higher number of individuals with diabetes and weak HGS (35).

## DISCUSSION

This systematic review identified 20 studies investigating associations between muscle strength measured with a handheld dynamometer and the occurrence of metabolic syndrome or some of its components in older adults. Most studies found consistent correlations between lower HGS and metabolic syndrome or its risk factors in older adults. Among metabolic syndrome components, high blood pressure was the only one that correlated with stronger handgrip in the older adults (24).

This qualitative synthesis indicates that low HGS in older adults is significantly associated with metabolic syndrome and with some of its components, except hypertension. In a recent nationwide cross-sectional study in Korea with 77,991 participants, high relative HGS was significantly associated with reduced risk of hypertension in adult and middle-aged (44). Thus, maintaining high relative HGS may be associated with protective benefits against hypertension in the long term. However, some studies included in this systematic review showed that a higher blood pressure was associated with higher HGS, especially in older diabetic men (29) and in very old Caucasian subjects (>85 years) (24). Evidence indicates that in the very old adults, higher blood pressure may be protective because it is associated with preservation of renal function (45), better cognition (46-48), and muscular strength, conditions that significantly depend on vascular function. The increased vascular resistance with aging was speculated to require greater blood pressure to maintain tissue perfusion and prevent further damage to ischemic peripheral organs, such as skeletal muscles (24,34).

The age-related decline in muscle mass and function is one of the most prevalent health problems in older adults, with a high rate of adverse outcomes (49). Pathological changes to this vital metabolically active tissue can profoundly affect older adults. According to the 2019 Sarcopenia Consensus, patients that have below-average HGS can be classified as "probable sarcopenia" and need further muscle evaluation to confirm the diagnosis. Sarcopenia has been associated with acute and chronic disease states, increased insulin resistance, fatigue, falls, and mortality (50,51), and is a powerful predictor of late-life disability (52,53). Metabolic syndrome and sarcopenia adversely affect the quality of life and contribute to increased frailty, weakness, dependence, morbidity, and mortality, all conditions that have been associated with aging and reduced HGS (6,18,54). Patients with metabolic syndrome and sarcopenia at the same time have a higher risk of severe health events than those with either metabolic syndrome or sarcopenia (55,56).

The mechanisms of muscle weakness associated with metabolic syndrome are not completely clear but are related to inflammation and insulin resistance (57). Recent evidence indicates a link between loss of muscle mass and insulin resistance and an increased prevalence of metabolic syndrome in adults with sarcopenia (19,58,59). Insulin resistance causes a reduction in glycogen and protein synthesis and an acceleration of protein degradation (58). Reduced skeletal muscle mass also contributes to insulin resistance, increases lipolysis, the release of free fatty acids from adipose tissue, and inhibits the growth hormone (GH)-insulin like growth factor 1 (IGF1) axis (60,61). Stessman and cols. investigated older adult patients with type 2 diabetes mellitus and found a consistent association between diabetes mellitus and low HGS (23). Previous studies have indicated that insulin resistance and alterations in glucose homeostasis are associated with decaying muscle strength (62-64). Insulin resistance is a central abnormality in the metabolic syndrome, and muscle mass and strength are strong protective factors independent of insulin resistance and abdominal fat accumulation (62,65). Therefore, current literature and the qualitative synthesis of this systematic review indicate that improving muscle strength may have wider advantages than previously appreciated regarding the attenuation of the impact of metabolic syndrome in the older adult population.

Older adults have a higher risk of developing sarcopenic obesity, a condition characterized by an important reduction in lean body mass associated with central obesity (55,66-68). Sarcopenic obesity has a greater impact on metabolic diseases and cardiovascular-associated mortality than either sarcopenia or obesity alone (9,69,70). The lipid overflow from the expanded adipose tissue leads to increased fat deposition in skeletal muscle, which may result in the development of muscle insulin resistance and a decrease in muscle mass (71-73). Moreover, visceral fat significantly increases the risk of insulin resistance, metabolic syndrome, and cardiovascular diseases (60,74). More recent data highlight abdominal obesity, as determined by waist circumference, as a cardiovascular disease risk marker that is independent of BMI (69). The five studies included in this review that used waist circumference as an outcome found significant correlations between abdominal obesity and low HGS in older adults (31,33,39,41,42). Finally, reduced HGS was also associated with dyslipidemia in three studies included; low HGS was associated with hypertriglyceridemia (37,42) and with low HDL levels (37,39). These results indicate that HGS may be a useful tool to detect sarcopenic obesity and metabolic syndrome in older adults (75).

The strength of this systematic review is in the broad search strategy adopted, which was conducted in several different databases. The main limitation was not conducting a meta-analysis owing to the heterogeneity of metabolic syndrome outcomes and data analysis of the included studies. However, the qualitative synthesis of the included studies allowed us to conclude that most studies found a clear correlation between low HGS and metabolic syndrome or some of its components, especially abdominal fat and insulin resistance.

In conclusion, most studies examined in this systematic review revealed significant correlations between reduced HGS and metabolic syndrome or some of its components in older adults, especially abdominal obesity and insulin resistance. These results corroborate previous findings that below-average HGS is a proper indicator of health outcomes in the older adult population and supports the use of HGS in clinical settings as a predictor of adverse outcomes related to metabolic syndrome in older people.

## SUPPLEMENTARY MATERIAL:

### 1) The full mapping terms for the search strategy divided by sectors.

For the population:

DECS terms: (Sarcopenia OR Sarcopenias OR "Força Muscular" OR "Fuerza Muscular" OR "Inibição Muscular Artrogênica") AND (Mãos OR mão OR Mano) OR ("Fuerza de la Mano" OR "Força da Mão" OR "Aperto de Mão" OR Empunhadura) AND (Idoso OR Idosos OR Idosas OR Idosa OR "Pessoa de Idade" OR "Pessoas de Idade") AND (Idoso OR Idosos OR Idosas OR Idosa OR "Pessoa de Idade" OR "Pessoas de Idade").

MESH terms: (Sarcopenia) OR (Sarcopenia) OR (Muscle Strength) OR (Strength, Muscle) OR (Muscle Inhibition) OR (Muscle Inhibitions) AND (Hand) OR (hands) / (Hand Strength) OR (Hand Strengths) OR (Strength, Hand) OR (Strengths, Hand) OR (Grip) OR (Grips) OR (Grasp) OR (Grasps) AND (Aged OR Elderly OR senescence OR aging).

For the interventions:

DECS terms: Dinamômetro de Força Muscular OR "Dinamómetro de Fuerza Muscular".

MESH terms: (Muscle Strength Dynamometer) OR (Dynamometer, Muscle Strength) OR (Dynamometers, Muscle Strength) OR (Muscle Strength Dynamometers) OR (Dynamometer) OR (Dynamometers).

For the outcomes:

DECS terms: "Síndrome Metabólica" OR "Síndrome Metabólico" / (Síndrome Metabólica) OR (Síndrome Metabólico).

MESH terms: "Metabolic Syndrome" OR "Metabolic Syndromes" OR "Syndrome, Metabolic" OR "Syndromes, Metabolic" OR "Metabolic Syndrome X" OR "Insulin Resistance Syndrome X" OR "Syndrome X, Metabolic" OR "Syndrome X, Insulin Resistance" OR "Metabolic X Syndrome" OR "Syndrome, Metabolic X" OR "X Syndrome, Metabolic" OR "Dysmetabolic Syndrome X" OR "Syndrome X, Dysmetabolic" OR "Reaven Syndrome X" OR "Syndrome X, Reaven" OR "Metabolic Cardiovascular Syndrome" OR "Cardiovascular Syndrome, Metabolic" OR "Cardiovascular Syndromes, Metabolic" OR "Syndrome, Metabolic Cardiovascular" / (Metabolic Syndrome) OR (Metabolic Syndromes) OR (Syndrome, Metabolic) OR (Syndromes, Metabolic) OR (Metabolic Syndrome X) OR (Insulin Resistance Syndrome X) OR (Syndrome X, Metabolic) OR (Syndrome X, Insulin Resistance) OR (Metabolic X Syndrome) OR (Syndrome, Metabolic X) OR (X Syndrome, Metabolic) OR (Dysmetabolic Syndrome X) OR (Syndrome X, Dysmetabolic) OR (Reaven Syndrome X) OR (Syndrome X, Reaven) OR (Metabolic Cardiovascular Syndrome) OR (Cardiovascular Syndrome, Metabolic) OR (Cardiovascular Syndromes, Metabolic) OR (Syndrome, Metabolic Cardiovascular) ((Metabolic Syndrome) OR (Metabolic Syndromes) OR (Syndrome, Metabolic) OR (Syndromes, Metabolic) OR (Metabolic Syndrome X) OR (Insulin Resistance Syndrome X) OR (Syndrome X, Metabolic) OR (Syndrome X, Insulin Resistance) OR (Metabolic X Syndrome) OR (Syndrome, Metabolic X) OR (X Syndrome, Metabolic) OR (Dysmetabolic Syndrome X) OR (Syndrome X, Dysmetabolic) OR (Reaven Syndrome X) OR (Syndrome X, Reaven) OR (Metabolic Cardiovascular Syndrome) OR (Cardiovascular Syndrome, Metabolic) OR (Cardiovascular Syndromes, Metabolic) OR (Syndrome, Metabolic Cardiovascular) AND (english[Filter] OR portuguese[Filter] OR spanish[Filter])) AND ((((Sarcopenia) OR (Sarcopenia) OR (Muscle Strength) OR (Strength, Muscle) OR (Muscle Inhibition) OR (Muscle Inhibitions)) AND ((Hand) OR (hands))) AND ((Muscle Strength Dynamometer) OR (Dynamometer, Muscle Strength) OR (Dynamometers, Muscle Strength) OR (Muscle Strength Dynamometers) OR (Dynamometer) OR (Dynamometers))) ((((Hand Strength) OR (Hand Strengths) OR (Strength, Hand) OR (Strengths, Hand) OR (Grip) OR (Grips) OR (Grasp) OR (Grasps)) AND (Aged OR Elderly OR senescence OR aging)) AND ((Muscle Strength Dynamometer) OR (Dynamometer, Muscle Strength) OR (Dynamometers, Muscle Strength) OR (Muscle Strength Dynamometers) OR (Dynamometer) OR (Dynamometers)) AND (english[Filter] OR portuguese[Filter] OR spanish[Filter])) AND ((Metabolic Syndrome) OR (Metabolic Syndromes) OR (Syndrome, Metabolic) OR (Syndromes, Metabolic) OR (Metabolic Syndrome X) OR (Insulin Resistance Syndrome X) OR (Syndrome X, Metabolic) OR (Syndrome X, Insulin Resistance) OR (Metabolic X Syndrome) OR (Syndrome, Metabolic X) OR (X Syndrome, Metabolic) OR (Dysmetabolic Syndrome X) OR (Syndrome X, Dysmetabolic) OR (Reaven Syndrome X) OR (Syndrome X, Reaven) OR (Metabolic Cardiovascular Syndrome) OR (Cardiovascular Syndrome, Metabolic) OR (Cardiovascular Syndromes, Metabolic) OR (Syndrome, Metabolic Cardiovascular) AND (english[Filter] OR portuguese[Filter] OR spanish[Filter])).

### 2) Critical appraisal tool to assess the quality of cross-sectional studies (AXIS)

Reference: AXIS, Appraisal Tool for Cross-Sectional Studies (Downes et al., 2016).

**Table:**

Table 1	Number of articles attending criteria
1. Were the aims/objectives of the study clear?	
2. Was the study design appropriate for the stated aim(s)?	
3. Was the sample size justified?	
4. Was the target/reference population clearly defined? (Is it clear who the research was about?)	
5. Was the sample frame taken from an appropriate population base so that it closely represented the target/reference population under investigation?	
6. Was the selection process likely to select subjects/participants that were representative of the target/reference population under investigation?	
7. Were measures undertaken to address and categorise non-responders?	
8. Were the risk factor and outcome variables measured appropriate to the aims of the study?	
9. Were the risk factor and outcome variables measured correctly using instruments/measurements that had been trialled, piloted or published previously?	
10. Is it clear what was used to determined statistical significance and/or precision estimates? (eg, p values, CIs)	
11. Were the methods (including statistical methods) sufficiently described to enable them to be repeated?	
12. Were the basic data adequately described?	
13. Does the response rate raise concerns about non-response bias?	
14. If appropriate, was information about non-responders described?	
15. Were the results internally consistent?	
16. 1Were the results for the analyses described in the methods, presented?	
17. Were the authors' discussions and conclusions justified by the results?	
18. Were the limitations of the study discussed?	
19. Were there any funding sources or conflicts of interest that may affect the authors' interpretation of the results?	
20. Was ethical approval or consent of participants attained?	

**Table 2:** AXIS score of the articles included in the qualitative synthesis

Study ID First author, year		AXIS score (max. 20)
Biblioni, 2018		16
Chen, 2021		16
Chun, 2019		18
Escribà-Salvans, 2022		17
Gong, 2022		17
Joo, 2022		17
Kawamoto, 2016		17
Kim, 2020		17
Kimura, 2021		10
Leite, 2023		16
Lin, 2021		16
Merchant, 2020		17
Pérez-Sousa, 2020		17
Sayer, 2007		19
Souza Moreira, 2022		20
Stessman, 2017		20
Taekema, 2011		17
Tong, 2022		16
Waqas, 2022		17
Zhang, 2021		18

### 3) Cohen's Kappa statistics

Results of the inter-rater evaluation of the first screening, comparing the agreement between the two pairs of raters.

% of agreement: 87.47913188647746%

Cohen's k: 0.48399434891974746

Moderate agreement
